# Prophylactic Intravenous Lidocaine at Different Doses for Fentanyl-Induced Cough (FIC): A Meta-Analysis

**DOI:** 10.1038/s41598-018-27457-3

**Published:** 2018-07-02

**Authors:** Wulin Tan, Si Li, Xiaochen Liu, Xiang Gao, Wenqi Huang, Junying Guo, Zhongxing Wang

**Affiliations:** 1 Department of Anaesthesiology, 1st Affiliated Hospital of Sun Yat-sen University, Guangzhou, Guangdong, P. R. China; 2 The Sixth Affiliated Hospital of Sun Yat-sen University, Guangzhou, Guangdong, P. R. China; 3 Zhongshan School of Medicine, Sun Yat-sen University, Guangzhou, Guangdong, P. R. China; 4 Department of Pharmacy, 1st Affiliated Hospital of Sun Yat-sen University, Guangzhou, Guangdong, P. R. China

## Abstract

To evaluate whether different doses of intravenous lidocaine are effective at preventing fentanyl-induced cough (FIC), we searched PubMed, Scopus, Cochrane Library, EMBASE and Web of Science, according to predefined criteria, for all articles published until June 2017. A meta-analysis and subgroup analysis were performed by combining the reported incidence of FIC. The odds ratio (OR) was used as a summary statistic. Eleven articles were included, with 965 patients in the lidocaine group and 745 patients in the control group. A pooled analysis indicated that the overall incidence of FIC was significantly different between the lidocaine group and the control group (OR, 0.27; 95% confidence interval (CI), 0.21–0.35; P < 0.05), as well as between the adult and paediatric subgroups. Sensitivity analysis showed that the results were stable. Subgroup analyses showed that compared to a placebo, both low (0.5–1.0 mg/kg) and high doses of lidocaine (1.5–2.0 mg/kg) were effective at reducing FIC incidence. There was no significant difference between low or high doses of lidocaine. Fentanyl doses added no significant heterogeneity as shown by meta-regression. The findings of this meta-analysis indicate that prophylactic intravenous lidocaine is effective at preventing FIC in both adults and children.

## Introduction

Fentanyl is one of the widely used opioids as a pre-induction aid due to its rapid onset, short duration of action, intense analgesia, cardiovascular stability, and low histamine release. However, coughing is one side effect of fentanyl, occurring in 28–65% of patients and raising concern among anaesthesiologists^1–4^. Fentanyl-induced cough (FIC) usually occurs within two minutes after fentanyl injection. Even though FIC is usually benign and brief, it can require immediate intervention in some circumstances^3,5^. FIC may be associated with an unexpected increase in intra-cranial, intra-ocular and intra-abdominal pressure^3,5^. Some researchers report that severe FIC could cause multiple conjunctival and periorbital petechiae^5^. In addition, explosive spasmodic coughing has been reported to cause massive engorgement of the tongue and hypopharynx, which can lead to acute airway obstruction and severe hypoxia in the paediatric population^6^.

These adverse cough reflexes during endotracheal operation can be suppressed by the intravenous administration of lidocaine^7^. A reported mechanism shows that lidocaine might be able to depress the function of the central brainstem or block tracheal and hypopharyngeal cough receptors^8^. The use of lidocaine for the prevention of FIC has been previously mentioned^6, 9–11^, but the dosage of lidocaine varied across different studies. As young age is a risk factor of FIC^12^, the effect of lidocaine has not yet been distinguished between children and adults. Therefore, we conducted a meta-analysis of randomized controlled trials (RCTs) to evaluate the efficacy of lidocaine at different doses and in different patient groups for the prevention of FIC.

## Results

### Study characteristics and quality assessment

**Figure 1:**
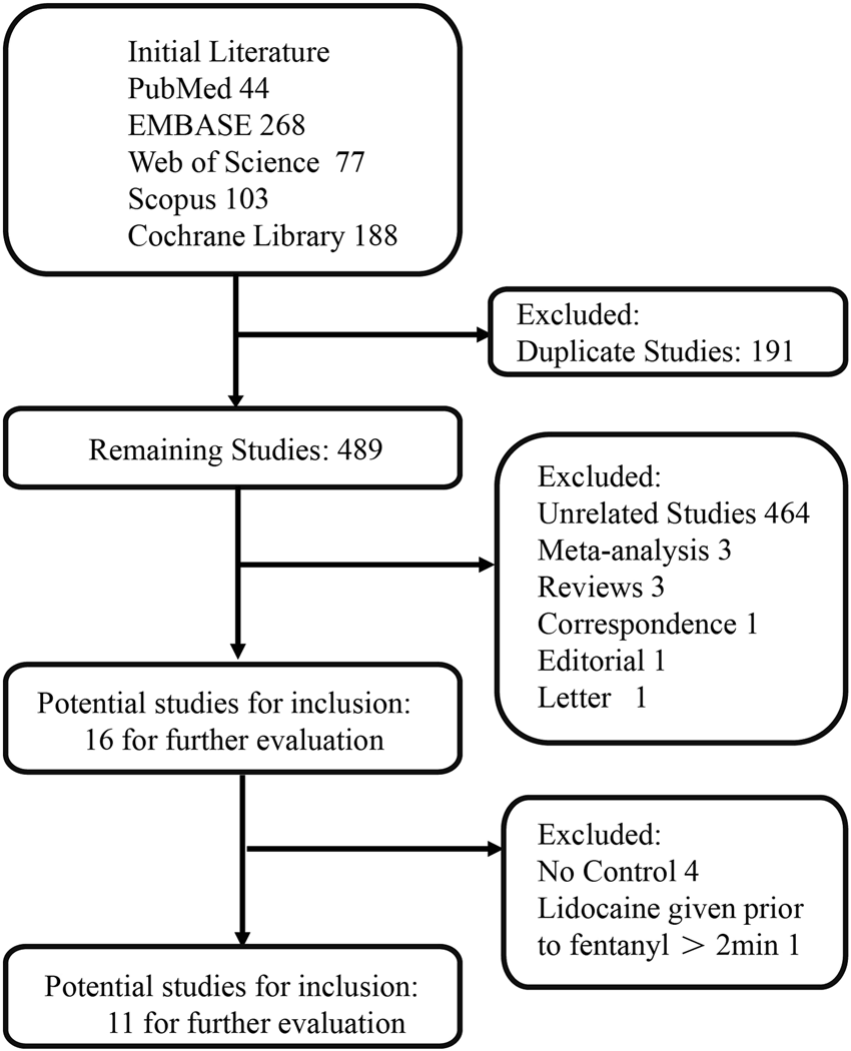
Search flow diagram.

Initially, our search strategy identified 680 articles. Approximately 191 studies were excluded because of duplication. In addition, 464 unrelated articles and other meta-analyses, reviews, correspondence, editorials, and letters were excluded according to our criteria. After further reading, 4 articles were excluded for not having a control group, and 1 RCT was excluded for the unmatched timeframe of lidocaine injection. Finally, we identified 11 full articles, with a total of 1710 patients, for detailed analysis. A flow diagram of the study selection process is presented in Fig. 1. In these studies, 965 patients in the lidocaine group were compared with 745 patients in the control group.

**Figure 2:**
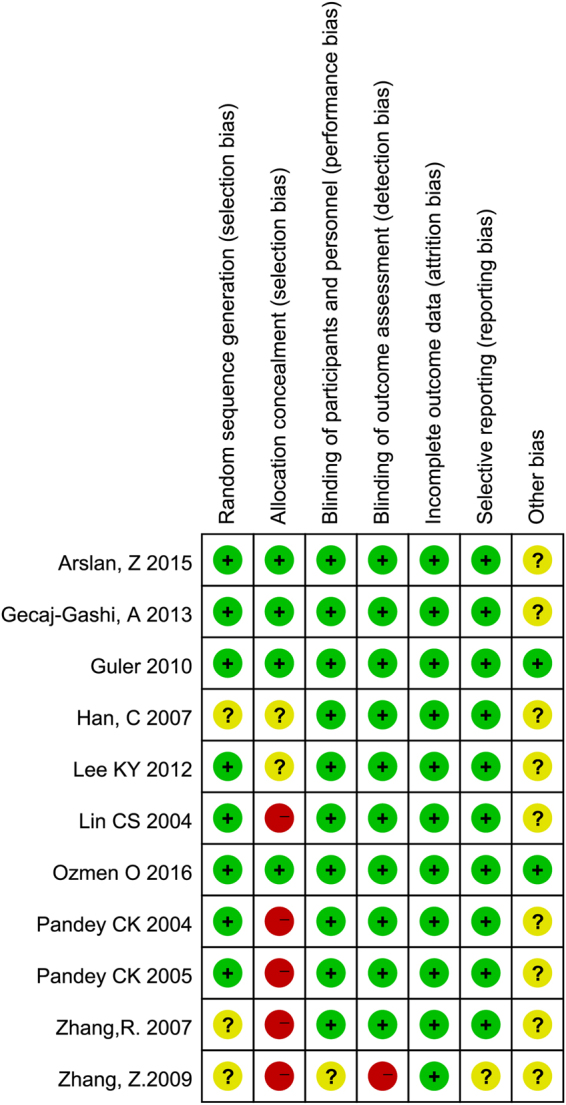
Risk of bias summary plot.

The study characteristics are summarized in Table 1. The risk of bias of these studies was assessed using Cochrane’s instructions. The methodological quality assessment is shown in Fig. 2. There was a high risk of bias among random sequence generation and allocation concealment assessments. Among all studies, 8 RCTs clearly described the method of random sequence generation, 10 RCTs clearly described the blinding of participants and personnel, and 10 RCTs clearly described the blinding of outcome assessments. One RCT did not describe the blinding of the observer, and the risk of detection bias was considered high. All the included studies clearly described incomplete outcome data. Only 4 studies provided enough detail for allocation concealment.

**Table 1:** Characteristics of the included studies.

Study	Year	Country	Blinding	Incidence of cough		Dose of fentanyl	Injection speed	Intervention (Lidocaine)	ASA grade	Age
				Lidocaine	Control					
Lin, CS^26^.	2004	Taipei, Taiwan	Observer	4/29	20/31	2.5 µg/kg	2 s	2 mg/kg	1~2	18–65
Pandey, CK^13^	2004	India	Observer	33/251	86/251	3 µg/kg	NA	1.5 mg/kg	1~2	18–60
Pandey, CK^14^	2005	India	Observer	34/240	28/80	3 µg/kg	NA	0.5, 1.0, 1.5 mg/kg	1~2	18–60
Han, C^31^	2007	China	Observer	4/25	12/25	4 μg/kg	3 s	1.5 mg/kg	1~2	20–60
Zhang, R^32^	2007	China	Observer	11/20	18/20	10 μg/kg	5 s	1.5 mg/kg	1~2	5–6
Zhang, Z^33^	2009	China	NA	10/30	18/30	3 µg/kg	3 s	1 mg/kg	1~2	18–65
Guler, G^34^	2010	Turkey	Operator; observer	11/100	23/100	2 µg/kg	Over 2 s	1 mg/kg	1~2	18–65
Lee, KY^27^	2012	Korea	Observer	9/66	35/66	2.5 µg/kg	NA	0.5 mg/kg	1~2	18–64
Gecaj-Gashi, A^7^	2012	Kosova	Observer	24/124	27/62	2–3 µg/kg	NA	0.5, 1.0 mg/kg	1~2	4–10
Arslan, Z^35^	2016	Turkey	Operator; observer	6/40	15/40	5 µg/kg	5 s	1 mg/kg	3~4	≥18
Ozmen, O^36^	2016	Turkey	Operator; observer	3/40	8/40	2 µg/kg	3 s	1 mg/kg	1~2	18–65

NA = Not available.

### Assessment of the incidence of FIC

**Figure 3:**
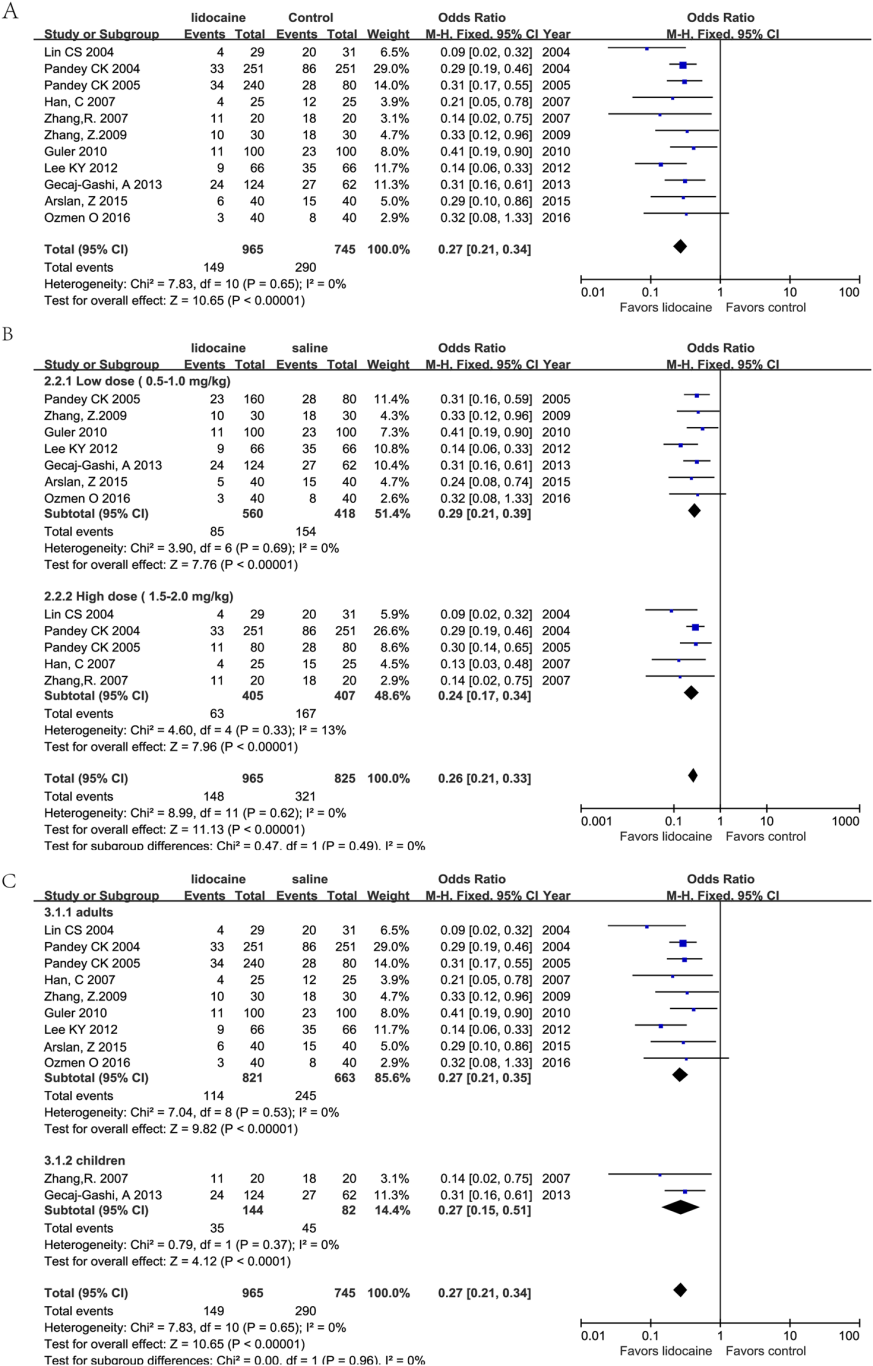
Forest plots for the effects of lidocaine on FIC. (**A**) lidocaine vs placebo. (**B**) Subgroup analysis for different doses of lidocaine. (**C**) Subgroup analysis in adults and children.

Eight of the included RCTs evaluated the incidence of FIC within 2 minutes after fentanyl injection, while 3 studies^7,13,14^ did not describe the time interval for cough observation. The results of this meta-analysis indicated that the incidence of FIC was significantly lower in the lidocaine group than in the control group (odds ratio (OR) = 0.27, 95% confidence interval (CI), 0.21–0.34, P < 0.05). There was no significant heterogeneity (*I*^2^ = 0%) (Fig. 3A). A subgroup analysis was implemented for different lidocaine doses (low dose, 0.5–1.0 mg/kg and high dose, 1.5–2.0 mg/kg). Based on our results, the incidence of FIC decreased significantly for both high and low doses of lidocaine (OR = 0.29, 95% CI 0.21–0.39, *I*^2^ = 0%, *P* = 0.69 and OR = 0.24, 95% CI 0.17–0.34, *I*^2^ = 0%, *P* = 0.33, respectively) (Fig. 3B). In addition, sensitivity analysis indicated no significant difference when excluding the RCT with high detection bias.

### Subgroup analyses

Subgroup analyses in adults and children showed a significant decrease in FIC incidence in both subgroups (OR = 0.27, 95% CI 0.21–0.35, *I*^2^ = 0%, *P* = 0.53 and OR = 0.27, 95% CI 0.15–0.51, *I*^2^ = *0%*, *P* = 0.37, respectively) (Fig. 3C).

Meta-regression with fentanyl dose as a covariant showed no significant heterogeneity (*P* > 0.05, slope CI [−0.28, 0.14]).

**Figure 4:**
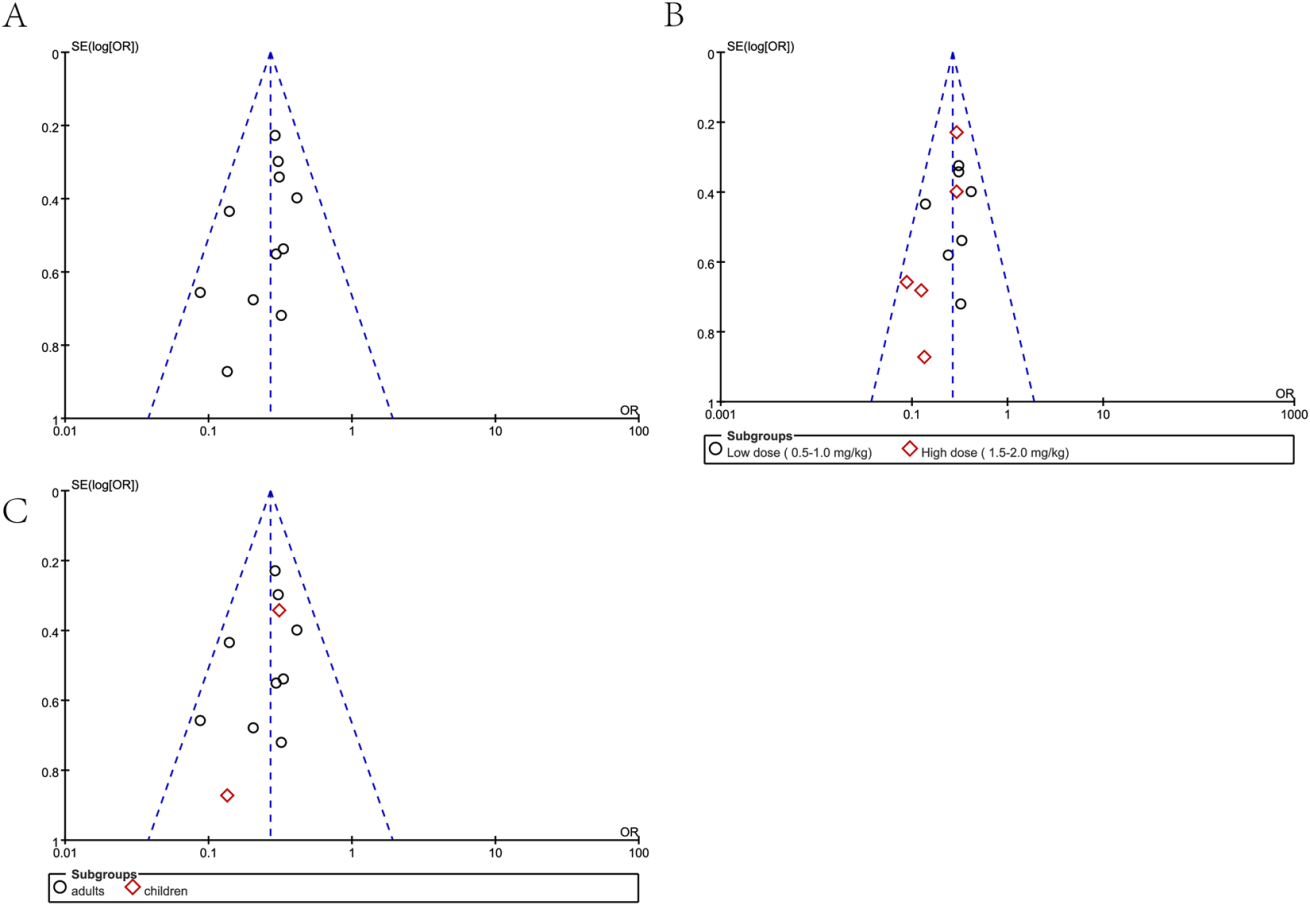
Funnel plots for the effects of lidocaine on FIC. (**A**) Comparison between the lidocaine and saline groups. (**B**) Subgroup comparison for different doses of lidocaine. (**C**) Subgroup comparison in adults and children.

Based on the quality of the RCTs included in this analysis, the strength and summary of evidence were further evaluated by GRADEpro 3.6.1, a statistical tool provided by the Cochrane Collaboration (Table 2). Asymmetric funnel plots (Fig. 4) suggested the existence of publication bias in our outcomes. With the addition of a high risk of detection bias in one RCT, these biases downgraded the outcomes of the grading. Qualitatively, the results of this review may be considered reasonably low.

**Table 2:** Grading of recommendation assessments, development and evaluation (GRADE) evidence profile for lidocaine use in fentanyl-induced cough (FIC) (using GRADEpro, version 3.6.1).

**Comparison between lidocaine and saline for preventing FIC**					
**Patient or population:** Adults and children scheduled for various elective surgeries under general anaesthesia with FIC. **Settings:** Hospital operating rooms. **Intervention:** Lidocaine intravenous injection. **Comparison:** Saline intravenous injection.					
Outcomes	Illustrative comparative risks* (95% CI)		Relative effect (95% CI)	No. of participants (studies)	Quality of evidence (GRADE)
	Assumed risk	Corresponding risk			
	**Placebo**	**Lidocaine**			
**FIC occurrence** Odds ratio	**Study population**		**OR 0.27** (0.21 to 0.34)	1710 (11 studies)	⊕⊕ ⊝ ⊝ **low** ^1 2^
	**389 per 1000**	**147 per 1000** (118 to 178)			
	**Moderate**				
	**436 per 1000**	**173 per 1000** (140 to 208)			

*The basis for the **assumed risk** (e.g., the median control group risk across studies) is provided in footnotes. The **corresponding risk** (and its 95% CI) is based on the assumed risk in the comparison group and the **relative effect** of the intervention (and its 95% CI).

**CI:** Confidence interval; **OR:** Odds ratio.

GRADE Working group grades of evidence.

**High quality:** Further research is very unlikely to change our confidence in the estimated effect.

**Moderate quality:** Further research is likely to have an important impact on our confidence in the estimated effect and may change the estimate.

**Low quality:** Further research is very likely to have an important impact on our confidence in the estimated effect and is likely to change the estimate.

**Very low quality:** We are very uncertain about the estimated effect.

^1^One RCT had a high risk of detection bias.

^2^Asymmetric funnel plots indicated the existence of publication bias.

## Discussion

The mechanism of FIC has not been well established, although various hypotheses have been proposed. Some studies^3,8,9^ attempt to explain FIC as follows: 1) stimulated by fentanyl, rapidly adapting receptors in airway mucosa cause bronchoconstriction; 2) C fibres on airway smooth muscles are stimulated by fentanyl which is a kind of citrate salt. Then these fibres release neuropeptides to cause cough. 3) histamine released by the mast cell in the respiratory system.

Even though the mechanism of FIC has not yet been clarified, the prevention of FIC is still meaningful. Although FIC can be transient and benign in some cases, in other cases, it can be severe^5,15^. FIC may cause an increase in the intra-cranial, intra-ocular, or intra-abdominal pressure, thus causing a series of severe complications during the induction of anaesthesia, such as ruptured cerebral aneurysms, regurgitation and aspiration, and worsening acute glaucoma^9^. In addition, patient groups with potentially increased intra-cranial pressure, acute glaucoma, serious airway responsiveness and penetrating eye injuries should be protected from FIC^9^. Particularly, in infants, who are highly vulnerable to FIC^8,16^, the prevention of this side effect may be very meaningful.

According to our meta-analysis, lidocaine can significantly reduce FIC incidence, consistent with previous studies^10,17^. Sensitivity analysis suggested a stable result. In addition, subgroup analysis demonstrated that lidocaine can prevent FIC in both adults and children. And intravenous lidocaine was also reported with safety and tolerability on pediatric patients to relieve pain^18,19^. Thus, we advocate that we can also apply lidocaine on children to prevent FIC. We included more studies than previous investigations^6,17^ and performed further analyses of the effect of lidocaine on FIC using different subgroups. However, the Grading of recommendation assessments, development and evaluation (GRADE) scoring for the evidence quality was not ideal, which was seldom mentioned in previous studies. Therefore, we suggest description of more details about the synthesized evidence when the results are reported.

Nevertheless, some confounding factors still need to be considered. According to previous reports, the incidence and degree of FIC seemed to vary based on several factors, such as the dose, the speed of injection and the route of administration. In our study, the included RCTs used different fentanyl doses. Higher doses of fentanyl might have a potential effect on a higher incidence of FIC^8^. Although fentanyl dose added no significant heterogeneity in the meta-regression, this may also be one of the confounding factors and should be considered when assessing the effect of lidocaine on FIC.

Even though the results of our study showed that both low and high doses of lidocaine might be beneficial for FIC, some RCTs reporting higher doses of fentanyl tended to use higher doses of lidocaine. However, the study by Pandey CK *et al*.^13^ showed that variable doses of lidocaine seemed to have similar effects on FIC with a constant dose of fentanyl, which is consistent with the results of our meta-analysis.

Intravenous lidocaine is widely used in some preoperative situations to reduce injection pain^20^ or to attenuate hemodynamic response during intubation^21–23^. In addition, adverse effects, including thrombophlebitis, sinus bradycardia, and dizziness, have been reported in previous studies^24,25^. In the study by Lin CS *et al*.^26^, there was one patient with dizziness and one with nausea and vomiting. In the study by Lee KY *et al*.^27^, the lidocaine group had lower mean arterial blood pressure than the control group but no significant difference in the incidence of dizziness (*P* > 0.05). Arrhythmia, hypotension and thrombophlebitis were not observed in the included RCTs. However, the incidence of these adverse effects is rare even with a dose as high as 2 mg/kg or a total of 100 mg for adults^28,29^. Individual practitioners may wish to use higher doses of lidocaine in the highest risk patients, by which the risk of adverse effects of lidocaine does not outweigh the possible benefit of preventing FIC.

## Conclusion

We conclude that prophylactic intravenous lidocaine, whether at low or high doses, is effective for preventing FIC in both adults and children.

## Methods

### Search strategy

We performed a systematic search of PubMed, Scopus, Cochrane Library, EMBASE, and Web of Science through June 2017 for relevant studies on the prevention of FIC by the prophylactic intravenous administration of lidocaine. The following subject terms and key words, including MeSH terms, were used in the search: (1) lidocaine, (2) fentanyl-induce cough, and (3) fentanyl cough. The search strategy was “lidocaine AND (fentanyl-induce cough OR fentanyl cough)”. The search was restricted to studies in human beings but not limited by language. To identify all potentially available articles, the references from relevant articles were also reviewed.

### Selection criteria

The titles, abstracts, and full texts of identified articles were reviewed. The included studies met the following criteria: (1) prospective RCTs, (2) patients receiving intravenous fentanyl, (3) prophylactic intravenous lidocaine vs placebo or no intervention, (4) lidocaine was given prior to fentanyl within 2 minutes, and (5) FIC incidence was the outcome. Studies were excluded if (1) patients presented with an obvious cough or upper airway responsiveness before receiving fentanyl, (2) patients had taken any other medications that may have influenced cough, and (3) the article reported any study design other than an RCT.

### Data extraction

The following information was collected from each study: first author, year of publication, sample size, age, American Society of Anaesthesiologists (ASA) classification, interventions and outcomes. The primary outcome was incidence (and odds) of cough in the lidocaine versus control groups. The secondary outcomes compared these findings between adult and paediatric populations. All included studies were independently scanned by two authors (Wulin Tan, Si Li). Discrepancies were resolved via review of the original articles and group discussion. A third author was consulted if the disagreement still existed.

### Statistical analysis

First, a meta-analysis was performed by combining the reported incidences of FIC in the lidocaine group and the control group. ORs and CIs were used to summarize the results. When I^2^ ≥50%, heterogeneity was considered moderate to high, and a random effects model was employed. The results were displayed in forest plots. The stability of results was detected by a sensitivity analysis. Publication bias was evaluated by funnel plots.

A subgroup analysis was performed to assess the effect of lidocaine on FIC at different doses (low dose 0.5–1.0 mg/kg vs high dose 1.5–2.0 mg/kg) and in different patient groups (adults vs children).

Meta-regression was conducted to evaluate whether fentanyl dose as a covariant contributed to heterogeneity^30^.

All statistical analyses were performed with Review Manager version 5.3. Meta-regression was performed in the open source software R.
